# A rare case of spinal involvement in hereditary hemorrhagic telangiectasia

**DOI:** 10.1038/s41394-024-00662-1

**Published:** 2024-07-17

**Authors:** V. Hvingelby, Ronni Mikkelsen, Gudrun Gudmundsdottir, Marlene Andersen, Ellen Merete Hagen

**Affiliations:** 1 West Danish Center for Spinal Cord Injury, Viborg, Central Region Denmark; 2 Department of Clinical Medicine, Aarhus University, Aarhus, Denmark; 3 Department of Neuroradiology, Aarhus University Hospital, Aarhus, Denmark; 4 Department of Biomedicine, Aarhus University, Aarhus, Denmark; 5 Department of Neurosurgery, Aarhus University Hospital, Aarhus, Denmark; 6 Institute of Neurology, University College London, London, UK

**Keywords:** Spinal cord diseases, Cerebrovascular disorders

## Abstract

**Introduction:**

Here, we describe a rare case of a spinal arteriovenous fistula in a patient with known hereditary hemorrhagic telangiectasia (HHT) and spontaneous intraspinal hemorrhage. Furthermore, we provide a brief review of the literature on the formation of spinal arteriovenous malformations (AVM) in relation to this disease.

**Case presentation:**

The case involves a 54-year-old male with known HHT. At the age of 49, the patient experienced recurrent cystitis. Urological evaluation ruled the cause to be neurological and subsequent imaging revealed a thoracic AVM. Four years later, the patient was admitted to A&E with chest pain and loss of function of the lower extremities and right arm, suspicious for ruptured aortic dissection. Trauma-CT excluded this and a final diagnosis of ruptured spinal AVM was made. Seven months post-injury, a spinal angiography was performed confirming the AVM. The remaining AVM was embolized under general anesthesia with acceptable results.

**Discussion:**

Spinal involvement in HHT is exceedingly rare but remains an important differential diagnosis, especially when patients present autonomic symptoms as these could potentially progress to life-threatening complications. The literature and the presented case indicate the prudence of closing spinal AVMs in HHT in case of symptoms, including autonomic, such as bladder dysfunction.

## Introduction

Hereditary hemorrhagic telangiectasia (HHT) is a rare hereditary disorder, inherited in an autosomal dominant pattern, previously known as Osler–Weber–Rendu syndrome [1]. Depending on regional differences, prevalence of the syndrome has been reported to be between 1:8000 to 1:5000, and thus classified as an orphan disease [2, 3].

Two distinct phenotypes (HHT1 and HHT2) are recognized and caused by mutations in several different genes [4]. While a multitude of genetic mutations have been identified leading to disease phenotypes similar to HHT, mutations in just two genes account for an estimated 90–98% of cases. Mutations on the endoglin gene (ENG) is the most common form (approximately 39–61% of cases) and leads to the HHT1 phenotype while mutations of the Activin A Receptor Type II-like 1 gene (ACVRL1) results in the less prevalent (approximately 25–57% of cases) HHT2. Common to both genes is that they encode endothelial membrane proteins involved in the quiescence pathway.

The diagnosis of HHT is made based on a positive family history and the presence of skin or mucosal nodules along with frequent and recurring epistaxis as the latter is present in upwards of 95% of cases [5].

Cases of life-threatening complications to HHT, while rarer than skin or nasal symptoms, are primarily caused by arteriovenous malformations (AVMs) developing in internal organs such as liver, lungs and central nervous system (CNS) and therefore warrant further interest. Here, we describe a case of HHT causing life-threatening complications due to a spontaneous hemorrhage of a spinal AVM. In addition, we review the published literature on spinal AVMs in the presence of HHT.

## Case presentation

A 54-year-old male was referred for specialized rehabilitation at the West Danish Center for Spinal Cord Injury, Denmark. The patient had no history of drug, alcohol or tobacco use. Previous medical history included known HHT since childhood and had received dermatological treatment for facial telangiectasias for cosmetic reasons. Due to recurrent cystitis, at the age of 49, he was referred for urological assessment, revealing incomplete bladder voiding as the probable cause. Urodynamic examination identified an overactive bladder with diminished detrusor function indicating a primary neurological cause. Therefore, the patient was referred to the Department of Neurosurgery together with a requisition of MRI of the neural axis. Neurological examination at this time was notable for brisk patellar reflexes and a slightly widened gait.

MRI revealed a spinal AVM at Th11 with dilated vessels extending along the cervical spine. Angiography identified a clear communicating fistula.

On examination, the patient had slight ataxia and hyper-reflexivity of the lower extremities. He had significant balance problems and marked fatigueability, presumably of neurological origin. Based on the clinical and imaging findings, operative closure of the fistula was recommended. However, in subsequent weeks, the symptoms subsided spontaneusly and patient declined further interventions at that time.

Four years later, at the age of 54, the patient was admitted to A&E with chest pain and loss of function of the lower extremities and right arm, suspicious for ruptured aortic dissection. Trauma-CT excluded this and the patient was referred for further neurosurgical assessment. Acute spinal MRI revealed edema and hematoma extending from C1 to Th12 with extinction of previously identified dilated vessels. Over the following weeks, the hematoma was partially resorbed in the absence of surgical intervention and spinal edema subsided partially. Three-month follow-up MRI of the neural axis revealed persistent intramedullary hemorrhage at Th3-6 and edema from C4-Th11.

Upon admittance for highly specialized spinal cord rehabilitation, patient exhibited paresis of the right upper and bilateral lower extremities. The International Standards for Neurological Classification of Spinal Cord Injury (ISNSCI) examination revealed a neurological level of C3 and AIS grade C, sensory level C3/C8 and motor level C3/C7 - incomplete tetraplegia. Neurologically, there was a complete sensorimotor deficit of the lower extremities. The right arm exhibited partial motor deficit with a grade of 3 at C6, 1 at C7 and 0 at C8 and T1. In terms of sensation, the right upper extremity likewise displayed a partial deficit with a complete absence of the ability to discriminate pin prick but preserved tactile sense at C5 and partially preserved at C6-T1. The left upper extremity had preserved sensorimotor function. Furthermore, the patient had no sensation of ampular filling and required daily laxatives, rectally. Since the patient had preserved the sensation of bladder filling at 300 mL, he was maintained on prophylactic antibiotics for recurrent cystitis.

A primary strategy of physiotherapy-guided rehabilitation was prescribed. Over the course of rehabilitation, three main challenges were identified. Firstly, the patient suffered persisting urological issues subsequent to spinal cord injury. Due to complete paralysis of the right hand and the patient being right-handed, he was unable to catheterize himself, leading to frequent bladder distention. This caused frequent cystitis even in the presence of prophylactic antibiotic treatment. During six months of rehabilitation, the patient was diagnosed with refractory cystitis on five occasions, including one episode of urosepsis. Importantly, the patient did not exhibit symptoms of cystitis other than urinary incontinence and on several occasions increased spasticity of the lower extremities prior to fulminant cystitis. Ultrasound four months post-injury revealed hydronephrosis of the right kidney without loss of function.

A long-term strategy of weekly alternating antibiotic prophylaxis of nitrofurantoin and pivmecillinam was pursued with an option of bladder surgery if insufficient.

The second challenge was the management of the persistent fistula.

**Fig. 1:**
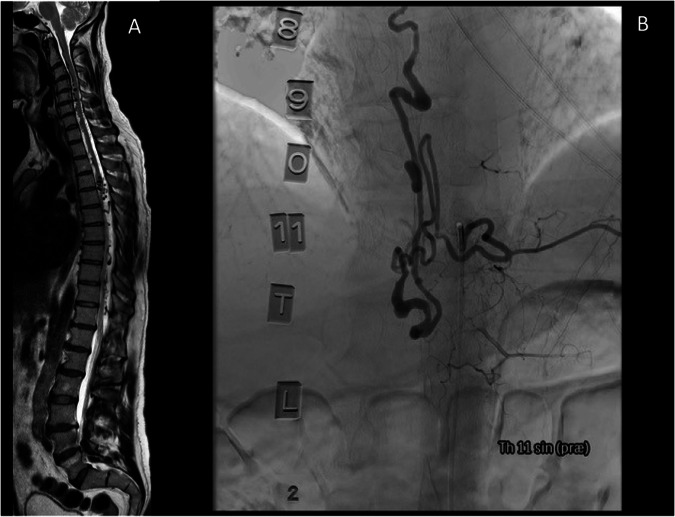
Magnetic resonance imaging (MRI) and angiography were performed prior to embolization of spinal arteriovenous malformation in our patient. Panel (**A**) shows an MRI sagittal image of the spinal column with multiple loci of tortuous vessels, particularly at levels Th4-Th6 and Th8-11. Image (**B**) of the panel shows the angiography performed prior to embolization.

**Fig. 2:**
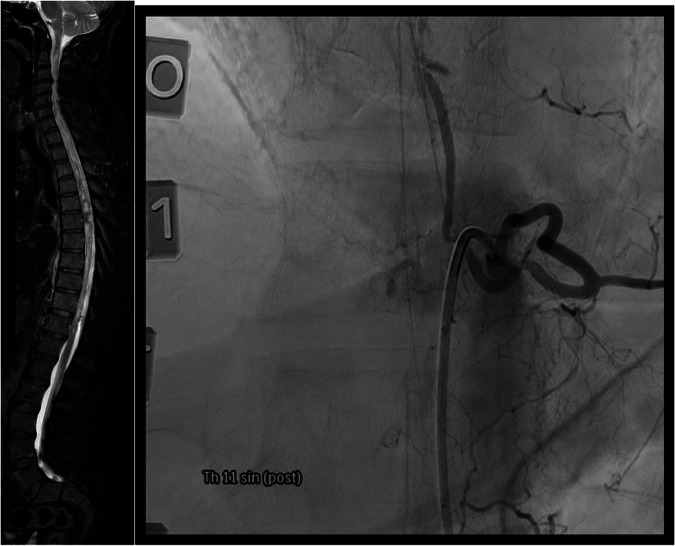
Similar to Fig. 1, Fig. 2 shows an MRI and angiography image of the same site, post-embolization. The MRI of the spine showing resolution of the lesions described previously and the angiography acquired subsequent to embolization, likewise, indicates clear resolution of the AVM.

Seven months post-injury, a spinal angiography was performed confirming the AVM (Fig. 1A, B). The remaining AVM was embolized under general anesthesia with acceptable results (Fig. 2A, B).

Upon admission for rehabilitation, the patients’ main presenting complaints were a nearly paralytic right upper extremity and paresis of the lower extremities. These complications caused disruptions to his rehabilitation. However, during rehabilitation, the function of his right triceps increased from MRC grade 0 to MRC grade 2 with full range of motion. Improvement of his hand function was limited. Dorsal flexion of the wrist was achievable, but no additional motor function of the hand could be facilitated. In the lower extremities, at the end of rehabilitation, dorsiflexion of the toes was attained bilaterally. Therefore, the patient remained wheelchair bound at discharge. At the latest follow-up patient remained wheelchair bound and persistently recurrent cystitis.

## Discussion

Here, we have presented a rare and highly complicated case of spinal cord injury caused by HHT in a middle-aged individual. Spinal AVMs are rare in HHT, comprising less than 1% of AVMs [1]. However, they represent an important consideration in managing HHT patients. For one, as the present case shows, they can result in potentially life-threatening conditions such as spontaneous hemorrhage. Additionally, other symptoms due to space-occupying lesions such as radiculopathy and myelopathy may occur. Consequently, spinal involvement is an important differential diagnosis, even in seemingly unrelated symptoms. Indeed, in the present case, the initial complaint of our patient was recurrent cystitis due to incomplete bladder voiding.

HHT is a rare congenital disorder and the presence of spinal involvement is exceedingly rare and infrequently described in the literature. A search performed across the PubMed/MEDLINE database identified twenty references [6–25]. Seventeen were case reports or series (*n* < 15) [6–9, 11–16, 18–21, 23–25], of which ten comprised pediatric patients [8, 9, 11–14, 16, 21, 23, 24]. Three references were larger case series or cohorts [10, 17, 22]. However, these were either comprising cases of HHT in general and not necessarily cases with confirmed spinal involvement [10], or cases of spinal AVMs not necessarily caused by HHT [17, 22]. In total, 59 cases of spinal AVM in the presence of HHT were identified in this search. One series, based on internal screening of more than 800 HHT patients spanning nearly two decades, revealed only four confirmed cases of spinal AVMs (< 0.4%) [10]. Importantly, three of these were identified due to patients developing serious complications and only one discovered incidentally. However, when viewed as a proportion of all spinal AVMs, two larger cohorts, both spanning nearly two decades of cases, including 210 and 155 individuals, respectively, revealed a prevalence of HHT of 1.4–3.2% (*n* = 3 and *n* = 5, respectively) [17, 22], implying a risk ratio of 3.5–8 of developing a spinal AVM. Importantly, however, both of these latter case series included only symptomatic patients referred for specialized neuroradiological evaluation and -treatment of these spinal AVMs. Moreover, given the potentially large heterogeneity of autonomic symptoms potentially elicited by spinal AVMs, it is not certain that even symptomatic spinal AVMs would raise clinical suspicion of spinal involvement and may even be adequately managed without this knowledge. The true incidence of spinal AVM in the setting of HHT is therefore likely underreported even in large case series. Of all reported cases of spinal AVM in HHT in which data on age was presented (*n* = 32), the age range spanned from less than 1 month to 77 years old (median 8 years, IQR 41.5 years), 20 pediatric cases. Most commonly identified cases presented with acute, serious neurological deficits with only seven cases being discovered incidentally, of which only two could be considered asymptomatic. Most commonly, symptoms were due to compression of the spinal cord (75%) versus outright bleeding or hemorrhage (25%). Of the 21 cases in which clinical symptoms were reported, six (28.6%) reported a history of bladder dysfunction or urinary retention.

Summarily, spinal involvement in HHT is exceedingly rare but remains an important differential diagnosis, especially when patients present autonomic symptoms as these could potentially progress to life-threatening complications. The literature and the presented case indicate the prudence of closing spinal AVMs in HHT in case of symptoms, including autonomic, such as bladder dysfunction.
